# Effective Range of Percutaneous Posterior Full-Endoscopic Paramedian Cervical Disc Herniation Discectomy and Indications for Patient Selection

**DOI:** 10.1155/2017/3610385

**Published:** 2017-10-26

**Authors:** Hongquan Wen, Xin Wang, Wenbo Liao, Weijun Kong, Jianpu Qin, Xing Chen, Hai Lv, Thor Friis

**Affiliations:** ^1^Department of Spinal Surgery, The First Affiliated Hospital of Zunyi Medical College, Zunyi, Guizhou 563000, China; ^2^Institute of Health and Biomedical Innovation, Queensland University of Technology, Brisbane, QLD 4059, Australia; ^3^Academy of Orthopedics of Guangdong Province, Department of Orthopedic Surgery, The Third Affiliated Hospital of Southern Medical University, Guangzhou, Guangdong 510630, China

## Abstract

The objective was to investigate the effective and safe range of paramedian CDH by percutaneous posterior full-endoscopy cervical intervertebral disc nucleus pulposus resection (PPFECD) to provide a reference for indications and patient selection. Sixteen patients with CDH satisfied the inclusion criteria. Before surgery the patients underwent cervical spine MRI, and the distance between the dural sac and herniated disc was measured. An assessment was performed by MRI immediately after surgery, measuring the distance between dural sac and medial border of discectomy (DSMD). The preoperative average distance between the dural sac and peak of the herniated disc (DSPHD) was 3.87 ± 1.32 mm; preoperative average distance between dural sac and medial border of herniated disc (DSMHD) was 6.91 ± 1.21 mm and an average distance of postoperative DSMD was 5.41 ± 1.40 mm. Postoperative VAS of neck and shoulder pain was significantly decreased but JOA was significantly increased in each time point compared with preoperative ones. In summary, the effective range of PPFECD to treat paramedian CDH was 5.41 ± 1.40 mm, indicating that DSMHD and DSPHD were within 6.91 ± 1.21 mm and 3.87 ± 1.32 mm, respectively. PPFECD surgery is, therefore, a safe and effective treatment option for patients with partial paramedian cervical disc herniation.

## 1. Introduction

Cervical disc herniation (CDH) is one of the most common degenerative spinal disorders and is characterized by upper extremity pain and neurological deficits. Depending on the site of the intraspinal disc herniation, CDH can be divided into three categories: median, paramedian, and lateral herniations [1]. There are several conservative treatments currently in use which have achieved therapeutic outcomes, such as medication with steroidal and nonsteroidal anti-inflammatory drugs and physical therapy; however, surgical interventions can be necessary for patients with severe cervical radiculopathy and myelopathy [2, 3]. Posterior laminoforaminotomy access to the cervical spine was developed in the early 1940s [4], whereas anterior access for the operation of cervical disc changes was described in the late 1950s [5]. Additional surgical approaches arising from posterior and anterior access have also been explored, such as anterior cervical decompression without fusion, anterior foraminotomy, posterior microscope-assisted or endoscope assisted “key-hole foraminotomy,” and cervical disc replacement [6, 7]. The anterior cervical discectomy and fusion (ACDF) procedure is commonly regarded as the most successful approach to CDH treatment since it can maximally attenuate herniated disc compression and maintain anterior stability [8, 9]. In most cases, these techniques will result in satisfactory patient outcomes; however, the ACDF procedure, owing to a high fusion rate, is considered the gold standard for the treatment of CDH [10, 11]. Given the high frequency of ACDF operations, there have been reports of complications stemming from this procedure, such as the formation of pseudarthrosis, severe degeneration of adjacent segments, height reduction of the intervertebral space (IVS), and motion loss of the cervical spine fusion [12, 13].

In an effort to reduce such surgery-related complications, there has been a push to develop advanced endoscopic techniques, of which full-endoscopic cervical discectomy (FECD) has been extensively used. FECD falls into two main categories: anterior (AFECD) and posterior (PFECD). AFECD is mostly used for patients with median CDH, which can effectively resect a protruded intervertebral disc to decompress the spinal cord. However, this is a risky approach given the proximity of major blood vessels and nerves [14]. Furthermore, damaged intervertebral disc tissues stemming from AFECD can lead to spinal instability and negatively impact postoperative recovery. By comparison, PFECD is generally considered a safer procedure for patients with the lateral CDH. One of the key advantages of PFECD is that it tends not to disturb the intervertebral disc [15, 16]. In this report, we demonstrate that PFECD can be used to treat paramedian CDH when a partially protruding nucleus pulposus, close to the front of the cord, is properly removed. The rationale for this study was to investigate the effective and safe range of paramedian cervical discectomy with percutaneous posterior full-endoscopy, which can serve as a reference for its surgical indications.

## 2. Materials and Methods

### 2.1. Patient Characteristics

Sixteen patients (seven female, nine male) were recruited for this study. The patients presented with paramedian CDH, as defined by a paramedian herniation that pressed the spinal cord unilaterally and deformed it into a comma shape, pressing the spinal cord and nerve root and then generating myeloradiculopathy symptom [1]. All the patients, whose ages ranged from 26 to 62 years (mean: 42 years), underwent percutaneous posterior FECD between August 2015 and September 2016. The reported duration of pain ranged from 1 to 78 months (mean: 13 months), and preoperative neurologic presentation included myelopathy in ten patients and myeloradiculopathy in six patients. The operations targeted levels C4-C5 in one patients, C5-C6 in six patients, and C6-C7 in nine patients.

### 2.2. Inclusion Criteria

The inclusion criteria were as follows: (1) having received failed conservative treatment lasting for more than 4 weeks or symptoms deteriorating to the extent of becoming unbearable; (2) neurological symptoms (myelopathy and/or myeloradiculopathy) consistent with the preoperative magnetic resonance image; and (3) single-level paramedian disc herniation. The exclusion criteria were as follows: (1) clear segmental instabilities or deformities; (2) cervical intervertebral disc with calcification; (3) isolated neck pain for which the cause could not be determined by magnetic resonance imaging (MRI); (4) foraminal stenosis without disc herniation; (5) multiple-level disc herniation; (6) previous surgery at the same segment; and (7) a suspected infection or tumor in the cervical spine.

### 2.3. Preoperative Evaluation

**Figure 1:**
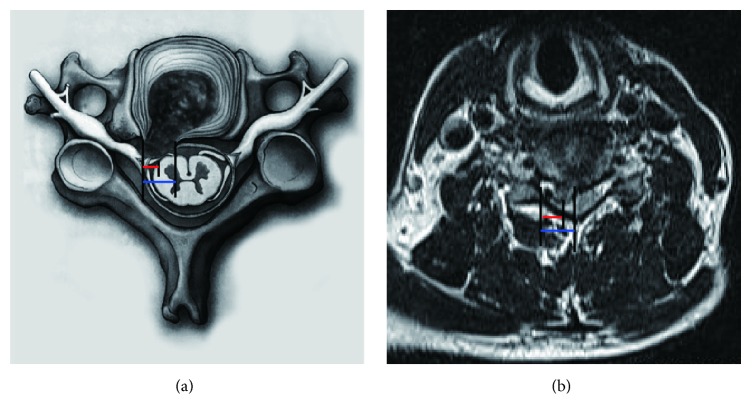
(a) The lateral border of dural sac and peak of herniated disc (DSPHD; red line) and lateral border of dural sac and intersection of dural sac and medial border of herniated disc (DSMHD; blue line) are shown in schematic picture. (b) DSPHD (red line) and DSMHD (blue line) are shown in MRI image.

The examinations were performed by two surgeons with experience in this technique. The vertical distance between the lateral border of the dural sac and peak of the herniated disc (DSPHD) and the distance between the lateral border of the dural sac and the intersection of the dural sac and medial border of the herniated disc (DSMHD) were recorded by two independent doctors from MRI images (Figure 1). The final values were calculated as the average of triplicate measurements from each doctor. In addition, the Visual Analog Scale (VAS) was used to determine neck and arm pain and the modified Japanese Orthopedic Association (JOA) scoring system to determine functional status.

### 2.4. Operative Technique

**Figure 2:**
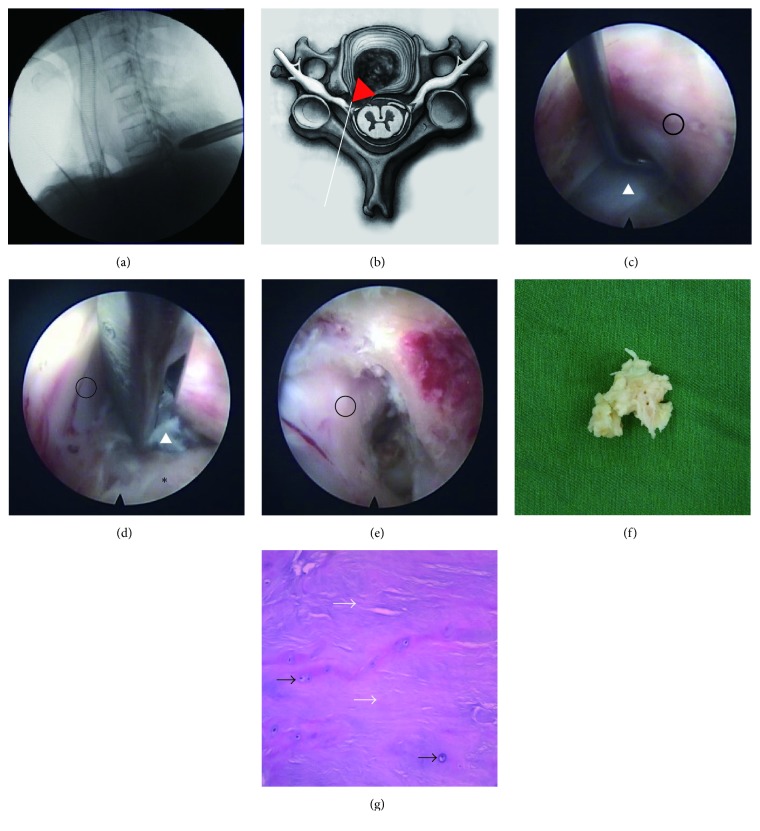
(a) Preoperative lateral radiograph image of a 42-year-old man with paramedian CDH at C6/7. (b) Endoscope sketch map showing a paramedian disc herniation. (c–e) Pre-, intra-, and postoperative view at C6/7 with dural sac (black circle), protrusive CDH (white delta), and vertebrae (black asterisk). (f) Protrusive cervical intervertebral disc tissue. (g) Histology of CDH with collagen (white arrows) and chondrocytes (black arrows).

Operations were performed under general anesthesia with the patients placed in a prone position with heightening at the chest to keep the neck flexed. The patients' shoulders were immobilized with tape and the arms placed caudally on the body with gentle tension to aid the fluoroscopic visualization of cervical levels. The line of spinal joints was marked using posterior-anterior radiography guidance, whereas the operations were guided by lateral radiography. Once the location of the cervical segment had been accurately determined, a skin incision was made and a dilator with a 6.9 mm outer diameter bluntly inserted into the facet joint. The operation sheath was inserted via the beveled opening of the operation performed under visual control and the site was irrigated continuously with 0.9% saline solution. The facet joint was completely exposed and grinded with a high-speed grinding drill. The lateral ligamentum flavum was resected to expand the intervertebral foramen to allow the endoscope to penetrate into the spinal canal, after which the herniated disc tissue was resected. The nucleus pulposus of the cervical intervertebral disc was ablated by radiofrequency (RF) ablation; the mobilization of the nerves root was repeatedly checked (Figure 2). Finally, all instruments were removed and the incision closed by suturing. Operation times, bleed volumes, and intraoperative complications for each patient were recorded.

### 2.5. Follow-Up

**Figure 3:**
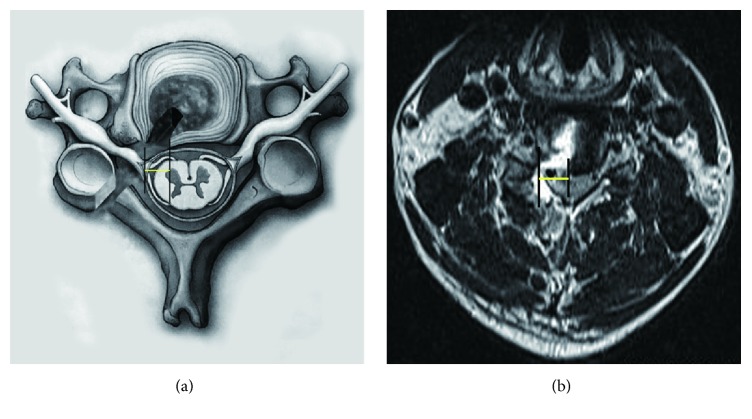
(a) The lateral border of dural sac and intersection of dural sac and medial border of discectomy (DSMD; yellow line) are shown in schematic picture. (b) DSMD (yellow line) is shown in MRI image.

All patients were followed up 3, 28, 90, and 180 days after surgery, each patient receiving a questionnaire by mail four working days ahead of their attendance at the clinic. The follow-up examinations were conducted by two physicians, neither of whom had been involved in the operations. Besides general parameters, other relevant information was collected using the following evaluations: the modified Macnab criteria were used to evaluate the postoperative outcomes, whereas VAS and JOA scores were recorded at the final follow-up visit [17, 18]. MRI scans were taken of each patient at day 3, and the distance between the lateral border of the dural sac and the intersection of the dural sac and medial border of discectomy (DSMD) were measured from the MRIs of the cervical spine in the transverse plane (Figure 3). These measurements were done in triplicate by two doctors who had not been involved with the operations, and the average value of these six measurements constituted the final data points.

### 2.6. Statistical Analysis

Statistical analysis was performed using the Statistical Package for the Social Sciences (ver. 18.0, SPSS, Chicago, IL, USA). The Tamhane test and Dunnett test were applied to compare pre- and postoperative VAS and JOA scores at various times. The differences between pre- and postoperative distance measurements were analyzed using a paired sample *t*-test. In all analyses, a probability < 0.05 was considered significant. Results were presented as a mean ± standard deviation.

## 3. Results

### 3.1. Perioperative Complications

None of the patients experienced any preoperative or postoperative complications, such as postoperative bleeding, injury to the nerve or dura, damage to the spinal cord with hemi-/paraparesis or paralysis of the upper extremities. There were not any complications from infection, spondylodiscitis, or thrombosis. Deterioration of existing symptoms was not observed in any of the patients.

### 3.2. Clinical Outcomes

Preoperative DSPHD and DSMHD were estimated at 3.87 ± 1.32 mm and 6.91 ± 1.21 mm, respectively. Postoperative DSMD was 5.41 ± 1.40 mm (Table 1). The VAS scores for neck pain at days 3 (3.20 ± 0.42), 28 (1.40 ± 0.52), 90 (1.20 ± 0.42), and 180 (1.10 ± 0.24) after surgery showed that patients experienced significantly less pain and discomfort over time compared with the preoperative VAS (8.10 ± 0.88) (*P *< 0.05). The decrease in VAS, when comparing postsurgery day 3 with days 28, 90, and 180, were also statistically significant (*P *< 0.05). However, there was no significant difference between VAS scores recorded at days 28, 90, and 180 (*P *> 0.05) (Table 2).

Cervical spinal function as measured by the JOA score showed an increase from 8.90 ± 0.74 before operation to 13.20 ± 0.42 at day 3, 14.50 ± 0.53 at day 28, 14.80 ± 0.42 at day 90, and 15.10 ± 0.62 at day 180 after operation, a statistically significant difference (*P *< 0.05). The difference in JOA score at day 3, when compared with JOA scores at days 28, 90, and 180, was significant (*P *< 0.05), whereas the differences amongst the JOA scores at days 28, 90, and 180 failed to reach significance (*P *> 0.05) (Table 2). Applying the modified Macnab criteria [19] to evaluate the curative effect 6 months after surgery, 13 cases were deemed to be excellent and 3 cases deemed to be good.

## 4. Discussion

Surgical intervention represents the most efficacious clinical alternative for CDH cases that fail to respond to conservative treatments. An operation by a surgeon can effectively relieve the spinal cord or nerve root compression and promote the recovery of its function, thus achieving a significant improvement in clinical symptoms [19–21]. In recent years, the application of minimally invasive surgical techniques has reduced many of the unfavorable factors associated with traditional open surgery, such as tissue trauma and excessive bleeding and considerable risk of nervous, parenchymal, and vascular lesions which was associated with an increased hospital stay [22–24]. Additional benefits include reduced recovery time in bed after surgery and lower incidents of severe complications, such as hypostatic pneumonia or deep venous thrombosis of the lower limbs. There is less impact on the muscles and nuchal ligaments attached to the vertebral plate and spinous process via sequential dilation, which would otherwise need to be isolated during the operation [22, 25]. This results in reduced postoperative scar tissue formation which in the past could lead to persistent pain and discomfort in the back of the neck and a faster return to work [7, 26, 27].

With percutaneous posterior endoscopic cervical intervertebral disc nucleus pulposus resections, the inner margin of the articular process is grinded with an abrasive drill to open a hole into the spinal canal. This greatly reduces damage to posterior ligaments, muscles, and bone, while retaining maximal biomechanical stability of the cervical vertebrae. A follow-up study of 87 patients found that two years after receiving percutaneous PFECD (PPFECD) operations, 87.4% of the patients reported no recurrence of neck or shoulder pain, and only 9.2% experienced occasional pain. Although the decompression results of PPFECD were similar to conventional ACDF, the operation-related traumatization was reduced [28]. PPFECD is now considered a safer and more effective treatment for cervical intervertebral disc herniation when compared with conventional ACDF and has advantages when compared with the anterior percutaneous endoscopic cervical intervertebral disc nucleus pulposus extirpation operation (PAFECD), such as reduction of the volume of disc removal, the length of hospital stays, and the postoperative radiographical changes [29]. The latter procedure requires the surgeon to go through the intervertebral disc, leading to unavoidable damage to the intervertebral disc which can cause issues such as postoperative accelerated disc degeneration, cervical instability, and loss of physiological flexibility. A two-year follow-up study of 103 patients having received APECD found that up to 12% of the patients had significantly decreased intervertebral height, increased incidence of cervical kyphosis, and occasional arm pain [30]. By comparison, with PPFECD there was no intervertebral disc damage and less damage to the surrounding muscles, ligaments, and zygapophysis due to the use of an intraoperative channel through the back of the neck. The result was no aggravation of cervical kyphosis deformity or postoperative decrease in intervertebral space height [31]. In the present study, sixteen patients underwent PPFECD surgery to remedy paramedian cervical disc herniation. All sixteen operations were successful and proceeded without any complications. The curative effects were deemed to be satisfactory, as measured by all patients reporting an absence or significantly reduced postoperative neck pain, eliminating the need for oral analgesics by the end of a 6-month follow-up study.

Previously, PPFECD has been applied to CDH patients whose symptoms included radiculopathy with upper extremity numbness and pain [16]. Since the use of this technique is less common to treat multisegmental CDH, we limited our study to patients with single segmental cervical intervertebral disc herniation. By applying a gentle and intermittent surgical procedure, we could remove the cervical intervertebral disc protruding from the inner side of the dural sac and compressing the spinal cord; this further expanded the resection range. In the observation period following the operation, symptoms of numbness and fatigue of one side of the body and upper and lower limbs and walking instability improved significantly. Our results indicate that PPFECD is an effective treatment option for spinal disorders caused by CDH which offers significant scope to investigate the resection range of PPFECD.

We selected the edge of dural sac as a position marker for primary reasons: (i) it has a clear boundary which makes it a good point of reference and (ii) the dural sac is rarely affected by the surgery so that risk of damage to the spinal cord is negligible. The removal of the cervical intervertebral disc protruding from inside the dural sac can increase the risk of spinal cord injury, due to stretching of the sac. Consequently, one of the key objectives of this study was to explore the range of resection possible with the PPFECD technique on cervical intervertebral disc herniating from inside the dural sac.

In the postoperation follow-up, we found that the distance between the edge of the dural sac and the inside edge of the intervertebral disc was significantly smaller than between the edge of the dural sac and the inside edge of the herniated disc. In other words, the resected amount of actual intervertebral disc tissues was less than that of the preoperative measurements. A possible explanation for this could be that the nucleus pulposus, not otherwise protruding from the disc, was RF ablated, causing a decrease in the pressure inside the intervertebral disc thereby retracting the remaining herniated disc. Postoperative symptoms of all patients were improved at all cervical levels, and cervical MRIs showed no evidence of protrusions compressing the spinal cord or nerves in any of the patient. At the final 6-month follow-up, there were no reported complications, such as spinal cord injury or dural sac rupture. Based on the analysis of all the experimental data, we propose that the application of PPFECD for cervical disc herniation up to 6.91 ± 1.21 mm and peak of herniated disc up to 3.87 ± 1.32 mm is safe and effective and that the safe resection range of cervical intervertebral disc using PPFECD is up to 5.41 ± 1.40 mm within the border of the dural sac.

In our study, all patients recovered fully from the operation, experiencing no serious or even minor complications and, most importantly, preoperative symptoms of pain, numbness, and fatigue were completely relieved at the six-month endpoint of the study. There were no reported symptoms of spinal cord injury, such as sensory disturbance, muscle weakness, pathological reflex, or defecation incontinence. PPFECD offers advantages such as smaller incisions, less tissue damage, and adequate nerve root decompression which result in faster postoperative recovery, with fewer complications, shorter hospital stays, and, therefore, reduced cost. These are benefits that point to PPFECD as a safe and effective surgical procedure for treating cervical disc herniation. With a more in-depth understanding of spinal anatomy and refinement of surgical techniques, the indication and application scope of PPFECD could be expanded even further, thus exploring new space for the treatment of paramedian type CDH.

## 5. Conclusion

In this study, we have found that the effective range was 5.41 ± 1.40 mm from the border of the dural sac for percutaneous posterior full-endoscopic cervical disc herniation discectomy treated paramedian cervical disc. For patients with partial paramedian cervical disc herniation was up to 6.91 ± 1.21 mm and peak of herniated disc up to 3.87 ± 1.32 mm within the lateral border of dural sac. PPFECD surgery is a safe and effective treatment option.

## Figures and Tables

**Table 1:** Pre- and postoperation measurements of distances between the dural sac and herniated disc and distances between the dural sac and the medial border of discectomy (mean ± SD).

Indicators	DSPHD (mm)	DSMHD (mm)	DSMD (mm)
Measured value	3.87 ± 1.32	6.91 ± 1.21	5.41 ± 1.40

DSPHD: the vertical distance between lateral border of dural sac and peak of herniated disc; DSMHD: the vertical distance between lateral border of dural sac and intersection of dural sac and medial border of herniated disc; DSMD: the vertical distance between lateral border of dural sac and intersection of dural sac and medial border of discectomy.

**Table 2:** Comparison of functional indicators recorded before percutaneous posterior full-endoscopic cervical discectomy (PFECD) and at last follow-up (mean ± SD).

Indicators	Pre-op	3 days	28 days	90 days	180 days
VAS score	8.10 ± 0.88	3.20 ± 0.42^*∗*^	1.40 ± 0.52^*∗*#^	1.20 ± 0.42^*∗*#△^	1.10 ± 0.24^*∗*#△*※*^
JOA score	8.90 ± 0.74	13.20 ± 0.42^*∗*^	14.50 ± 0.53^*∗*#^	14.80 ± 0.42^*∗*#△^	15.10 ± 0.62^*∗*#△*※*^

VAS score: homogeneity test of variance, *P* = 0.01 and *P* < 0.05, single factor analysis of variance using Tamhane test; JOA score: homogeneity test of variance, *P* = 0.22 and *P* > 0.05, single factor analysis of variance using Dunnett test; compared with preoperation, ^*∗*^*P* < 0.05; compared with postoperation day 3, ^#^*P* < 0.05; compared with postoperation day 28, ^△^*P*> 0.05; compared with postoperation day 90, ^*※*^*P* > 0.05.

## References

[1] Kokubun S., Tanaka Y. (1995). Types of cervical disc herniation and relation to myelopathy and radiculopathy. *Journal of Back and Musculoskeletal Rehabilitation*.

[2] Jho H.-D. (1996). Microsurgical anterior cervical foraminotomy for radiculopathy: A new approach to cervical disc herniation. *Journal of Neurosurgery*.

[3] Matsuda Y., Shibata T., Oki S., Kawatani Y., Mashima N., Oishi H. (1999). Outcomes of surgical treatment for cervical myelopathy in patients more than 75 years of age. *Spine*.

[4] Barr J. S. (1947). Ruptured intervertebral disc and sciatic pain. *The Journal of Bone Joint Surgery*.

[5] Cloward R. B. (1958). The anterior approach for removal of ruptured cervical discs. *Journal of Neurosurgery*.

[6] Nandoe Tewarie R. D. S., Bartels R. H. M. A., Peul W. C. (2007). Long-term outcome after anterior cervical discectomy without fusion. *European Spine Journal*.

[7] Hilton D. L. (2007). Minimally invasive tubular access for posterior cervical foraminotomy with three-dimensional microscopic visualization and localization with anterior/posterior imaging. *Spine Journal*.

[8] Saal J. S., Saal J. A., Yurth E. F. (1996). Nonoperative management of herniated cervical intervertebral disc with radiculopathy. *Spine*.

[9] De Rooij J. D., Gadjradj P. S., Huygen F. J., Luijsterburg P. A. J., Harhangi B. S. (2017). Management of Symptomatic Cervical Disk Herniation. *Spine*.

[10] Louie P. K., Presciutti S. M., lantorno S. E. (2017). There is no increased risk of adjacent segment disease at the cervicothoracic junction following an anterior cervical discectomy and fusion to C7. *The Spine Journal*.

[11] Hammer C., Heller J., Kepler C. (2016). Epidemiology and pathophysiology of cervical disc herniation. *Seminars in Spine Surgery*.

[12] Türeyen K., Maciejczak A. (2003). Disc height loss after anterior cervical microdiscectomy with titanium intervertebral cage fusion. *Acta Neurochirurgica*.

[13] Fountas K. N., Kapsalaki E. Z., Nikolakakos L. G. (2007). Anterior cervical discectomy and fusion associated complications. *Spine*.

[14] Tzaan W. C. (2011). Anterior percutaneous endoscopic cervical discectomy for cervical intervertebral disc herniation: outcome, complications, and technique. *Journal of Spinal Disorders & Techniques*.

[15] Ahn Y., Lee S. H., Chung S. E., Park H. S., Shin S. W. (2005). Percutaneous endoscopic cervical discectomy for discogenic cervical headache due to soft disc herniation. *Neuroradiology*.

[16] Ruetten S., Komp M., Merk H., Godolias G. (2008). Full-endoscopic cervical posterior foraminotomy for the operation of lateral disc herniations using 5.9 mm endoscopes: a prospective, randomized, controlled study. *Spine*.

[17] Yonenobu K., Abumi K., Nagata K., Taketomi E., Ueyama K. (2001). Interobserver and intraobserver reliability of the Japanese Orthopaedic Association scoring system for evaluation of cervical compression myelopathy. *Spine*.

[18] Zanoli G., Strömqvist B., Jönsson B. (2001). Visual analog scales for interpretation of back and leg pain intensity in patients operated for degenerative lumbar spine disorders. *Spine*.

[19] Yan D., Li J., Zhu H., Zhang Z., Duan L. (2010). Percutaneous cervical nucleoplasty and percutaneous cervical discectomy treatments of the contained cervical disc herniation. *Archives of Orthopaedic and Trauma Surgery*.

[20] Li J., Yan D.-L., Zhang Z.-H. (2008). Percutaneous cervical nucleoplasty in the treatment of cervical disc herniation. *European Spine Journal*.

[21] Jagannathan J., Sherman J. H., Szabo T., Shaffrey C. I., Jane J. A. (2009). The posterior cervical foraminotomy in the treatment of cervical disc/osteophyte disease: A single-surgeon experience with a minimum of 5 years' clinical and radiographic follow-up - Clinical article. *Journal of Neurosurgery: Spine*.

[22] Bonaldi G., Minonzio G., Belloni G. (1994). Percutaneous cervical diskectomy: preliminary experience. *Neuroradiology*.

[23] Wang M. C., Chan L., Maiman D. J., Kreuter W., Deyo R. A. (2007). Complications and mortality associated with cervical spine surgery for degenerative disease in the United States. *Spine*.

[24] Scoville W. B., Whitcomb B. B. (1966). Lateral rupture of cervical intervertebral disks.. *Postgraduate Medicine*.

[25] Schick U., Dohnert J., Richter A., Konig A., Vitzthum H. (2002). Microendoscopic lumbar discectomy versus open surgery: An intraoperative EMG study. *European Spine Journal*.

[26] DePalma A. F., Rothman R. H., Lewinnek G. E., Canale S. T. (1972). Anterior interbody fusion for severe cervical disc degeneration. *Surgery, Gynecology & Obstetrics*.

[27] Coric D., Adamson T. (2008). Minimally invasive cervical microendoscopic laminoforaminotomy. *Neurosurgical Focus*.

[28] Ruetten S., Komp U., Merk H., Godolias G. (2007). A new full-endoscopic technique for cervical posterior foraminotomy in the treatment of lateral disc herniations using 6.9-mm endoscopes: prospective 2-year results of 87 patients. *Minimally Invasive Neurosurgery*.

[29] Yang J.-S., Chu L., Chen L., Chen F., Ke Z.-Y., Deng Z.-L. (2014). Anterior or posterior approach of full-endoscopic cervical discectomy for cervical intervertebral disc herniation? a comparative cohort study. *Spine*.

[30] Ruetten S., Komp M., Merk H., Godolias G. (2009). Full-endoscopic anterior decompression versus conventional anterior decompression and fusion in cervical disc herniations. *International Orthopaedics*.

[31] Kim C. H., Shin K. H., Chung C. K., Park S. B., Kim J. H. (2015). Changes in cervical sagittal alignment after single-level posterior percutaneous endoscopic cervical diskectomy. *Global Spine Journal*.

